# A study on work-family life imbalance among women administrators in UAE higher education institutions

**DOI:** 10.1016/j.heliyon.2024.e28286

**Published:** 2024-03-16

**Authors:** Vazeerjan Begum, Tahseen Anwer Arshi, Abdelfatah Said Arman, Atif Saleem Butt, Surjith Latheef

**Affiliations:** a School of Business, American University of Ras Al Khaimah, Building 75, Sheikh Humaid Bin Mohammed Area, Seih Al Araibi, Ras Al Khaimah, 72603, United Arab Emirates; b Research and Community Service, Director of Entrepreneurship and Innovation, American University of Ras Al Khaimah, Building 75, Sheikh Humaid Bin Mohammed Area, Seih Al Araibi, Ras Al Khaimah, 72603, United Arab Emirates; c University of Queensland, Brisbane, Australia; d University of Westminster, UK

**Keywords:** Work-family imbalance, Higher education, Spillover theory, Work stress, Women administrators, UAE

## Abstract

The study explored the factors causing work-family imbalance among women administrators in higher education institutions in the UAE and how it affects their personal and organizational well-being. The research found that the existing literature doesn't give enough attention to the mismatch between women administrators' work and family goals. Furthermore, it provides little insight into the integration of work-family support systems. The study applied the Spillover theory to explain that women administrators face significant work-family imbalances that adversely impact their personal well-being and organizational effectiveness. The research also used Facilitation theory to examine how work-family support systems could reduce the adverse effects of work-family imbalances. The study surveyed 271 female administrators working in higher education institutions in the UAE. The findings, presented through structural equation modeling, showed that the demanding nature of research, teaching, and administrative work in higher education and women administrators' professional aspirations in socially demanding societies create work-life imbalance and work stress. The study proposed work-family support systems that could moderate the effect of work-family imbalances on work stress.

## 1 Introduction

Work-life balance (WLB) has emerged as one of the most significant challenges in today's modern organizations [1]. It refers to the primacy between an individual's professional and personal actions and the level of work-related activities present at home [2]. Work-life balance is “an equally satisfied level of involvement or ‘fit’ among the multiple roles in a person's life” (Konard and Mnagel [3]).

This study aims to explore the factors contributing to work-family life imbalance among female administrators in higher education institutions (HEIs). Specifically, the study investigates the unique challenges that arise from personal, professional, and social contexts that hinder women administrators from balancing work and family life. The study suggests that demands associated with higher education work, women's personal growth aspirations, and societal expectations can contribute to work-family life imbalances among female administrators. Furthermore, the study evaluates the impact of work-family life imbalance on female administrators' personal well-being and professional productivity. Finally, the study proposes organizational solutions to alleviate work-family imbalances experienced by women administrators in HEIs.

The literary trends developed around WLB are diverse. A bibliometric analysis of the corpus of WLB research by Rashmi and Kataria [4] revealed that four major thematic clusters are evident among the 945 research papers published between 1998 and 2020. These research patterns relate to gender discrimination in WLB, work-life interface tensions, and organizational policies and practices concerning WLB and work designs. Further, leading researchers in WLB, such as Kelliher et al. [1] and Casper et al. [5], argued that conceptualizing the central contracts in WLB research is not well aligned with the changing work patterns and demands in different industries. Casper et al. [5], through their meta-analysis of 290 WLB research papers, explained that most WLB research has focused on highlighting balance measures compared to work-life conflict measures.

### 1.1 Research background

The work-family imbalance for women administrators in higher education settings is a unique challenge, resulting from increasing career aspirations, work demands, and demanding family responsibilities.

The higher education sector is ideal for the study as the work demands are substantially diverse, involving quantitative and qualitative needs [6]. For example, several quantitative parameters apply to women administrators, such as research targets, student performance and satisfaction records, and administrative targets. Similarly, qualitative work demands require diverse skill sets to meet these work demands. According to Bakker and Demerouti [7] and Theron et al. [8], when quantitative work demands increase, a spillover effect is bound to create role conflict between work and home. These inter-role tensions and blurring of professional and personal boundaries lead to work-family spillovers, negatively impacting women administrators and their employers [9,10].

Naidoo-Chetty and Plessis [6] argued that organizational resources in higher educational institutions are insufficient to support women leaders’ career aspirations, and academic administrators are forced to utilize personal resources. Hartman and Barber [11] argued that women take a multifaceted approach to prepare for career success and build role competency. It requires them to dedicate themselves to developing leadership skills, and such commitments spill into their personal and social lives [12]. Shepherd [13] pointed out that career aspirations in the education sector are individualistic, and the onus of career development primarily resides with women leaders who are required to put in extra effort to reach and maintain leadership roles in HEIs. Further, Shaikh et al. [14] argued that senior managers' organizational commitment increases as they rise above the ranks, affecting their social and family time.

Mushfiqur et al. [15] argued that societal influences such as societal culture, societal demands, and societal support are crucial in creating work-life imbalances. Women administrators who seek to rise to higher positions must put in a considerable amount of work at the cost of family and social commitments [16]. The Arabic culture holds the stereotype that women should be homemakers, which puts pressure on women in administrative positions to fulfill both their family and societal obligations in addition to their work commitments. Although women are encouraged to work, they are still expected to prioritize their family responsibilities and fulfill societal commitments. According to Gragnano et al. [17], work-life imbalances extend beyond family demands and include health, lifestyle, leisure, and social relationships.

Work-family imbalances arising from the combination of these factors create work stress, low levels of well-being, lower morale, and low productivity at work [18]. Contrastingly, employers offering work-family supportive work environments enjoy long-term benefits such as employee retention, high-performance working employees, innovative work practices, and overall enhancement in their employer brand image [19].

### 1.2 Current research standing and research gaps

The extant literature on WLB is contextually weak and ontologically deficient [[20], [21], [22]]. Therefore, two prominent research gaps have emerged based on the studies conducted on WLB. These firstly relate to societal and industry contexts, which have not been well investigated. Secondly, most of the research on WLB has been viewed from an employee perspective, and the organizational viewpoints have not received adequate representation in WLB research.

The effect of rigorous work in higher education on work-life imbalance has not been explained well. Therefore, WLB research has yet to deal extensively with industry-specific WLB antecedents and support systems. For example, a higher education work environment characterized by the need for academic managers to remain engaged with research and teaching despite the demonstrative load, creating work-life imbalance, has yet to be adequately explored in the higher education sector [23]. Further, Delello et al. [24] and Denson et al. [25] pointed out that the effect of rigorous work on the quality of achievement of educational goals, teaching, and research performance has not been adequately analyzed. The rigorous workload of education, research, and administration hurts productivity and performance. As per the UAE Federal Authority for Government Human Resources Report [26], employee turnover rates are the highest among workers in the education sector. Parakandi and Behery [27] argued that work-life balance support systems are poorly examined in the Middle East, particularly in the United Arab Emirates. The report correlates that women administrators are finding difficulty striking an equilibrium between their professional and family lives in their academic administration profession. The extant literature identified several areas for further exploration within the selected geographic location. Firstly, minimal research has been done in the UAE on women's work-life imbalances in higher education. Secondly, the knowledge of the antecedents of the work-family life imbalance of women administrators in higher education institutions in the UAE is limited, as only a few research papers have been published [10,27]. Thirdly, the adverse impact of work-family imbalance on the personal and professional lives of women administrators must be evaluated to implement appropriate interventions.

Further, WLB research has focused on employee-perceived WLB factors and individual inability to deal with work-life imbalances; hence, sustainable solutions are scarce in WLB literature. The organizational solutions for WLB around policy development, procedures, and administration have not received adequate attention [28,29].

The HEI-specific work demands leading to work-family imbalances get more complicated under unique social conditions. Middle Eastern societies require higher social engagements and more significant family commitments than individualistic societies. Women administrators, for example, face work-life-imbalance issues due to family commitments and higher social engagements [30]. No research has considered the combined effect of challenging work conditions within a societal context in analyzing work-family life imbalances. Hence, there is a need to advance such research.

Based on these research gaps, this research aims to study the work-life imbalance of women administrators working in higher education institutions and develop a research model that can be tested and applied in the HEIs in the UAE. The study views work-life imbalances and resultant outcomes from an employee and organizational perspective. It underpins them with the Spillover and Facilitation theories to present a complete view of work-life imbalances in the higher education sector. Most importantly, it investigates them in socially demanding societies, highlighting how WLB can be disrupted when societal expectations outweigh work demands. Interestingly, it captures the tensions of work-life imbalances when socially pressing commitments clash with personal career aspirations.

## 2 Theoretical background

Researchers forwarded different theories to highlight the various aspects of Work-Life Balance [17,31]. Bello and Tanko [32] suggested that WLB theories should be selected based on the study's perspectives and framework. Therefore, the authors anchor the study to the spillover theory for several reasons. Firstly, women's connection to their families is emotionally deeper, profoundly affecting the spillover of work and family life (Taylor et al. [33]). As a result, the antecedents and outcomes of work-life imbalance assume greater construct validity. Secondly, Verfuerth et al. [34] identified that societal and environmental identity influences the spillover context. Therefore, the theory is well-suited as the adverse spillover effects are investigated in a socially demanding cultural context [35]. Spillover theory explains that inflexible and opaque social and work boundaries between work-family life are bound to create imbalances and increase the possibilities of work-family conflicts. The theory argues that when these domains are porous and flexible, integration between the domains is more manageable, reducing the chances of work-family conflicts. The theory suggests that a proper balance should be maintained between the segmentation and permeability among the domains [36]. However, in real situations, challenging work demands, emotional family life, and social commitments disturb this balance.

Further, the lack of WLB support systems creates an opaque and inflexible boundary between work and home. It leads to spillovers of activities, energies, and emotions, creating conflict between work and life domains. According to the Spillover theory, individuals carry feelings, attitudes, skills, and behaviors that they acquire and decide how to respond and react to a particular problem. When there is a negative spillover, difficulties, and depression in one domain may bring along the same emotions in another [37]. Deducing from these theories, one can argue that while the individual aspires to integrate and balance work and family life, circumstances are bound to create spillovers.

Further paying heed to Powell et al. [21], who argued there is a need for more theorizing, particularly for cross-fertilizing WLB theories, to underpin women-specific work-family imbalance models, this study integrated the spillover theory with the facilitation theory to derive focused work-family imbalance measures and moderating solutions to this enigmatic problem. Pollinating the two theories has provided an opportunity to bring an organizational perspective to the discussion on life imbalance, explore opportunities to reduce the adverse effects of the spillover theory, and give a better context and applicability to the facilitation theory. Facilitation theory is further suited to the study as it argues that individuals aspire for self-actualization, and organizations can empower employees to learn to strike a work-life balance. Additionally, organizations can facilitate learning and moderate the adverse effects of work-life imbalances by designing work environments that support work-life balance [38,39]. Moderating the spillovers, promoting work-life balance support systems, and reinforcing learning to adapt flexibility and self-awareness can play a crucial role. Identifying and testing an exhaustive list of WLB disruptors has occupied the research agenda for several years. Solution finding through the correct antecedents and moderators specific to women administrators' work-family imbalance framework should now drive the research priorities. Therefore, the cross-pollination of the two theories enabled the study to present a complete research framework for the study.

## 3 Literature review and hypothesis development

Work-life imbalance is a persistent issue influencing employees, their organizations, and the social fabric [19]. A good work-life balance can support women administrators in experiencing better handling of their work life (Wolor et al. [40]). In contrast, the work-life imbalance creates a depleting, unproductive, unaccommodating, and hostile workplace, signaling employees' insensitivity towards family life [41,42]. The extant literature shows that antecedents of work-family imbalance originate from work overload, unrealistic goals, personal ambitions, extended work timings, lack of energy, and new skills requirements. Various factors, including sector-specific work demands and societal conditions, can worsen the work-family imbalance. This can lead to work stress, characterized by low well-being and productivity. Studies by Adekoya et al. [19], Cassell [43], and Diego-Medrano et al. [44] have highlighted the negative impact of these antecedents on individuals.

### 3.1 Antecedents of work-life imbalance

Fisher-McAule*y* et al. [45] streamlined the WLB around three measurable dimensions: family life enhancement aspiration, work interference with family life, and family life interference with work. The dimensions reveal that the interference of work-related issues in family life and family issues in work-related issues are significant predictors of work stress. Similarly, Hayman [46] constructed a psychometric instrument for the measurement of the work-life balance of employees in organizations. A 15-item scale was designed after revision to record the employees' perceptions. It measured Work Interference with Family Life, Family Life Interference with Work, and Work-Family Life Enhancement as three constructs of work-life balance.

#### 3.1.1 Construct 1: work and family life enhancement

Work and family life enhancement aspirations relate to how job and private life enhance one another [6]. Women have career aspirations and ideal family goals and expect both to enrich each other (Sarwar et al. [47]). Work-life enhancement sub-factors facilitate comprehension of the support and enhancement provided by jobs in their family and family lives at work [18]. However, on the contrary, competing with their male counterparts on career aspirations at the expense of family time and commitment does not support work and family life enhancement. Many managerial-level women work extended hours to enhance career goals and enrich their careers, leading to work-family imbalances [48,49].

Further, work-life imbalances become more complicated when coupled with personal and professional growth aspirations and job enrichment. Naidoo-Chetty and Plessis [6] and Shaikh et al. [14] explained that women administrators' desire for professional advancement is challenging considering the need to excel in varied teaching, research, and administration areas, all requiring diverse skill sets. Therefore, individual characteristics, priorities, and job characteristics are the critical determinants of work-life imbalance. An individual's personality can be expressed based on two personality traits: workaholism and balance. Workaholism is considered an obsessive behavior when it is triggered by personal and professional aspirations [30]. The following hypotheses were constructed based on the discussion in the literature.

H1 Work-life enhancement aspirations will have a causal effect on the work-family imbalance

#### 3.1.2 Construct 2: work interference with family responsibilities

Hayman [46] states that work Interference with family responsibilities is related to job-related factors that impact an employee's family life. The nature of work for women administrators in higher education has changed over the years. The need for administrative skills must be supported by competencies in teaching and research [50,51]. Wilson et al. [9] and Agha et al. [10] argued that such a diverse set of work requirements requires additional time at work, and women administrators in higher education tend to carry work to home, which interferes with their family lives. The four sub-factors assessed of work Interference with family responsibilities are family life suffering because of work engagements, making family life challenging, placing family life on hold to fulfill job responsibilities, and needing work to catch up with deadlines [10]. The following hypotheses were constructed based on the discussion in the literature.

H2 Work-life interference in family life will have a causal effect on the work-family imbalance

#### 3.1.3 Construct 3: family responsibilities interference with work

According to Hayman [46], family responsibilities and interference with work are related to family factors that impact the job. It assesses the impact of private life on employees' work life. The determinants of work-life balance include family, individual characteristics, work demands, and technological advancements [52]. Family is an essential determinant of work-life balance. Any unfulfilled demand and expectation of the family can create significant interference between work and family life. In their study, Nizam and Kam [53] found that the determinants of work-life imbalance intensified in the absence of family support systems. The family domain's demands, especially in socially cohesive societies, include family responsibilities, commitments, family time, and role expectations [54]. Women, in particular, who have to bear the responsibilities of child care and elder care, suffer more from the problem of work-family conflict [55]. They juggle around their contending accountabilities like household chores, children, office work, partners, elderly parent care, and kinsfolks, which stresses the domestic, official, and societies in which they dwell [54,56]. The four sub-factors examined in family responsibilities interference with work are family life leaving little energy for work, tiredness affecting job effectiveness, work effectiveness reduction because of family life, and work becoming increasingly challenging because of family matters [30]. The study constructed the following hypotheses based on the literature.

H3 Family life interference in work life will have a causal effect on work-family imbalance

### 3.2 Outcomes of work-family life imbalance

Wang et al. [57] pointed out that the work-family imbalance has instigated bodily and psychological implications for women employees. The job demands are getting rigorous in the face of shrinking organizational resources, developments of technology, and global competition. Therefore, organizations expect a significantly higher level of commitment from employees. As a result, every employee must fulfill the rigorous demands of work and organization, requiring time, effort, and mental energy [58]. The impact of work-life imbalance on work stress, employee productivity, and burnout has been discussed by Yadav and Sharma [59] and Jackson and Fransman [60]. The negative impact of work-family conflict leads to high work stress and overall employee satisfaction and motivation [61,62]. Liu et al. [52], Bataineh [63], and Darko-Asumadu et al. [64] found that work-life imbalance had a significant impact on female employees' organizational commitment and also affected employee's mental health and well-being.

Sometimes, excessive work demands pose a significant threat to an individual and become a reason for stress and negative feelings about work, ultimately leading to low levels of well-being. Long working hours and work schedules are the most crucial factors leading to work stress [65]. Stress is the most common outcome of work-life imbalance and is an essential obstacle to employee productivity [66]. Persistent employee incompetence in managing their job and personal liabilities has significant negative consequences for the organization. Employees with poor WLB tend to be disengaged with work and the environment, decreasing work productivity [67]. The following hypotheses were constructed based on the discussion in the literature.

H4 Work-family imbalance will have a causal effect on work stress, leading to lower well-being and productivity levels.

### 3.3 The moderating role of work-family support systems

Organizations need to develop WLB support systems to improve employees' organizational commitment and loyalty, especially for senior administrators [17]. 2020). Organizational work patterns and demands have become multifaced and agile. Cuéllar-Molina et al. [68] examined the mediating impact of work-life balance and the role of human resource practices. One initiative is to create and manage work-life balance support systems by drafting WLB policies that provide the required freedom, time, and opportunities to balance their work and life. Shaikh et al. [14] emphasized retaining senior management teams through specified procedures that enhance organizational commitment. Tunio et al. [69] further supported the argument that human capital is critical in HEIs, and creating trust can help develop sustainable human resources practices focused on WLB. Similarly, Arabeche et al. [70] argued that organizations could create a culture supporting organizational performance and productivity. Therefore, corporate culture based on trust and employee and organizational commitment can support WLB practices. There are a few recommended work-life balance support systems, some of which are as follows.

#### 3.3.1 Attention management training

Companies create better work-life balance for employees by teaching attention management skills. Organizations train their talents and enhance their capacity to accomplish a particular work, enhancing their focus and maximizing their competency and skills. Althammer et al. [71] found that attention management training promoted psychological detachment between work and family lives and promoted active well-being, reducing stress at work. The outcome of attention management training is that the employees develop the skill to focus on work, maximize their productivity, and learn to relax and refresh during their free hours away from work [72].

#### 3.3.2 Support vacation-friendly culture

Companies instill the belief in the minds of their employees that there will be little work piled up after vacation return and that there is a substitute to handle issues in their absence. Employees take off from work to refresh and recharge themselves. Vacations enhance creativity and innovative perspectives in employees' minds, and many UAE HEIs promote this system through mid-semester breaks [73]. Furthermore, Brosch and his colleagues illustrate the significance of work engagement and well-being for vacation-related well-being attainments and the essential of work engagement in maintaining the positive effects of vacation [74].

#### 3.3.3 Policies on after-work hour's communication

One of the significant post-pandemic effects most women administrators face is the work hours spilling over to family time. To reduce this effect, organizations need help putting clear policy guidelines that restrict and state communication hours within the organization. Organizations only encourage employees to send emails or communicate after office hours in an emergency [75]. Kim and Chon [76] and Rendon [77] examined the effects of after-hours work communication through communication technologies on workers' burnout and added-role behaviors. The findings revealed that technological reach and flexibility have led to the spillover of official tasks over family space. It is also a reason for more variance between work and family time. Due to the pervasiveness of technology, employees face the challenge of enjoying the family life they have worked so hard to achieve [78].

#### 3.3.4 Hybrid work arrangement

One of the most critical post-pandemic changes in many organizations' work cultures is the shift to hybrid working arrangements. Organizations have realized that their talents work happily and exhibit high performance during flexible work periods. Many organizations provide employees with a hybrid work arrangement as part of the work-life support initiative. Researchers concur that the employees' morale tends to be high with higher control over their working conditions [79]. The work-life support initiative aims to improve stability between the stresses of the job and, as a result, excellent management of life outside the workplace and a diverse work environment. The review of the literature showed that research and insights into the work-family imbalance of women in Arab countries, especially in higher education institutions and in leadership positions, are minimal. The following hypotheses were constructed based on the discussion in the literature.

H5 A work-family life support system can moderate the relationship between work-life imbalance and work stress.

## 4 Research methodology

The study adopted a realist philosophy as its objective was to measure the work-life imbalance and work-stress phenomena as they occur. Therefore, a quantitative research method was adopted, allowing measurement of the phenomenon without much interference from probing and getting reliable, valid, and replicable results, as Bell et al. [80] suggested. At the same time, the study adopted a cross-sectional research design and developed a questionnaire survey to collect primary data from a sufficiently representative sample [81].

### 4.1 Sample

The purposeful sampling method was applied as only women administrators were selected for the study. These female administrators in the various higher education institutions in the UAE were chosen after an initial email request to be part of the survey was accepted by 325 female administrators. The questionnaire survey was initially piloted with 20 senior administrators after refinement of two items related to work-life enhancement and two items from work stress constructs. Once the face and content validity of the measures was established, the questionnaire survey was administered through the Google survey form. The survey form clearly described the study's objectives and the research questions. The response rate was reasonable as 280 respondents completed the survey, of which 271 were found fit for analysis. This number of responses is considered adequate for this research study as only a limited number of participants are available within the UAE higher education institutions [81].

### 4.2 Data collection and testing

The primary data collection instrument, the questionnaire survey, contained 5-point Likert-type questions to investigate the work-life balance issues of women administrators working in HEIs. Considering this is survey research, ensuring the validity of the latent constructs was critical for assessing the internal consistency and convergent validity of your measurement model. Therefore, the study utilized the congeneric approach in estimating factor loadings, as suggested by Marzi et al. [82]. A congeneric approach allowed better representation between the latent constructs and their indicators and reduced potential concerns related to the reliability of sum scores. It allowed adjustments of indicators on each item's unique loadings and error variances [83]. The study used a GLC estimator to compute latent variables. Finally, structural equation modeling tests using IBM SPSS version 22 and AMOS version 21 were conducted to test the research model and hypothesize a moderating relationship, as Tabachnick and Fidell [84] suggested.

### 4.3 Measures

The study utilized several established measures to examine work-life balance and work stress. First, work-life balance was measured using the work-life balance scale developed by Agha [85]. The work-life balance scale was reflected through primary constructs: Work Interference with Family Life, Family Life Interference with Work, and Work and Family Life Enhancement. The goodness of fit indices for all constructs was >0.90, and scale reliability for all measurement items was also >0.90 except for work-life enhancement, which was 0.88. The work stress scale was adapted from the Work Stress Scale developed by Torvisco et al. [86]. The 25-item scale developed based on World Health Organization and International Labor Organization guidelines contained 25 items, of which anxiety and adverse effects on health and well-being were assessed in this study. The scale reliability for all measures showed Cronbach alpha scores >0.80 with a replicability >0.80.

## 5 Data analysis

The study analyzed the data using various quantitative tests in line with its quantitative focus. Firstly, the internal reliability of the measures was checked through Cronbach Alpha scores, setting the benchmark >0.70. Next, correlation tests were conducted to examine the direction and strength of the relationship between the hypothesized variables. The reliability test of the interval scaled data for the factorial structure of work-life balance, work stress, and work-life balance support system indicators showed good internal consistency as Cronbach's alpha values were >0.70 (Table 3). Further, the author examined the validity of the measures to ensure that face, construct, and discriminant validity are established.

The data was subjected to statistical scrutiny before applying the structural equation modeling tests. Based on Tabachnick and Fidell's [84] recommendations, evidence for heteroscedasticity was checked to ensure that the sample across the educational institutions remained relatively high in their composition, which might have harmed their opinion. The homogeneity of variances in Levene's test showed that the sample showed characteristics of homoscedasticity. Levene's statistic resulted in a score of > 0.05 and single column Tukey HSD on all demographic factors such as number of years in the organization, number of years in the education sector, and size of the organization. Further, the authors checked for evidence of multi-collinearity through variance inflationary factor (VIF) scores to ensure that the independent variables did not inflate the correlation values. The results of the VIF test showed values < 2.5 and tolerance levels <10, ruling out multi-collinearity effects.

The Pearson correlations matrix in Table 1 showed positive correlations between the latent variables, except Family Life Interference in Work Life and Work Interference in Family Life (−0.368, p < 00.001). The highest correlation was evident between Work Interference in Family Life and work stress (0.698, p < 00.001).

**Table 1:** Mean, standard deviation, and Pearson correlations matrix.

Variable	Mean	Std Dev	WE	PLWI	WPIL	WLBS	WS
Work-Family Life Enhancement (WLE)	4.117	0.712	–				
Family Life Interference in Work Life (PLWI)	4.183	0.617	−0.549**	–			
Work Interference in Family Life (WFIL)	4.111	0.621	0.574**	−0.368**	–		
Work and Family Support System (WFSS)	3.917	0.715	0.498*	0.584**	0.587**	–	
Work Stress (WS)	4.210	0.618	0.497*	0.512**	0.698**	0.412**	–

n = 271, *p < 0.05; **p < 0.01; ***p < 0.001.

An SEM-based measurement model was developed and tested to check the convergent and discriminant validity of the constructs and associated indicators. Per the recommendations of Marzi et al. [82] and McNeish & Wolf [83], the data was further analyzed through a congeneric approach, which helped improve the reliability and validity of measured latent constructs, namely Work Life enhancement, Family Life Interference in Work Life and Work Interference in Family Life, Work Life Balance Support and Work Stress. The maximum likelihood and weighted average methods were used to estimate each factor's scores in the CLC estimator. The CLC estimator output showed the Cronbach alpha and Average Variance Extracted scores according to their weights. The assigned loadings were based on item-construct correlation values-the higher the item-construct correlation, the higher the weight of the assigned loadings. The average factor loadings showed values > 0.7, which meet the standards of established benchmarks [87]. In addition, the Average Variance Extracted (AVE) scores for all variables were >0.5, showing convergent validity. Finally, the fit indices were according to the established benchmarks. The chi-square indicates the model fit between the hypothesized model and measurement items. A good model fit was achieved as the results showed a low chi-square value relative to the degrees of freedom (2.78, df 2.29). The root mean square error of approximation was common, 0.04, the incremental fit indicator Tucker-Lewis index was recorded as 0.95, and the comparative fit index showed a value of 0.97. The data from the congeneric model are summarized in Table 2.

**Table 2:** Factor loadings, alpha scores, and average variance extracted (AVE) scores.

Variables and their Scale Items	Factor Score	Cronbach Alpha	AVE
Work-family Life Enhancement		0.79 (0.71)	0.694
1 I am aware that sometimes, I sacrifice my family life to achieve my career goals	0.80		
2 I am ambitious about my career aspirations and need family support	0.78		
3 I want to enhance my family life through the right balance between work and family	0.75		
Work Interference in Family Life		0.80 (0.78)	0.741
1 My family life suffers because of my workload towards research, teaching and administrative work	0.81		
2 I feel social pressure as my work makes it difficult to fulfill social obligations	0.79		
3 I put my family life on hold to fulfill job responsibilities	0.77		
4 I am not able to catch up with my family engagements because of work	0.74		
Family Life interference with work		0.81 (0.79)	0.705
1 My family life leaves me with little energy for work	0.81		
2 I am too tired to be effective in my job	0.79		
3 My emotional connection towards family takes priority over work commitments	0.75		
4 It is challenging to work because of family commitments	0.73		
Work balance support system		0.75 (0.71)	0.655
1 My organization has adequate measures to improve the work-family life balance	0.81		
2 My organization has policies to accommodate my family life requirements	0.79		
3 I receive adequate managerial support to compensate for my work deadlines due to family life commitments	0.70		
Work Stress		0.76 (0.70)	0.638
1 My work and family life create anxiety and ill-being	0.75		
2 I feel irritated even with minor issues	0.73		
3 My work and family life are affecting my work productivity	0.71		
4 My energy levels are drained, which affects my well-being	0.70		

n = 271, Kaiser-Meyer-Olkin (KMO values in parentheses).

**Table 3:** Hypotheses testing results.

Hypothesis Testing	Model Fit	Coefficient value	Result
H1*: Work-life enhancement aspirations will have a causal effect on the work-family imbalance*	Good	0.72***	Accept
H2*: Work-life interference in family life will have a causal effect on the work-family imbalance*	Good	0.78***	Accept
H3*: Family life interference in work life will have a causal effect on work-family imbalance*	Good	0.73***	Accept
H4*: A work-family imbalance will have a causal effect on work stress*	Good	0.76***	Accept
H5*: A work-family life support system can moderate the relationship between work-life imbalance and work stress.*	Good	0.68***	Accept

The complete research model was tested through a formative model per the study's hypotheses. The causal measures tested if ‘work-family life interference,’ ‘family life work interference,’ and ‘work-family life enhancement’ variables impacted work-life imbalance. The hypotheses about the formative measurement of a work-life imbalance were confirmed with coefficient values > *0.70 (0.72; 0.73; 0.78, p* < *0.01).* The results showed that work-family enhancement (*0.72, p* < *0.001)*, family life interference in work life *(0.73, p* < *0.001)*, and work interference in family life *(0.78, p* < *0.01)* significantly impacted work-life imbalance.

**Fig. 1:**
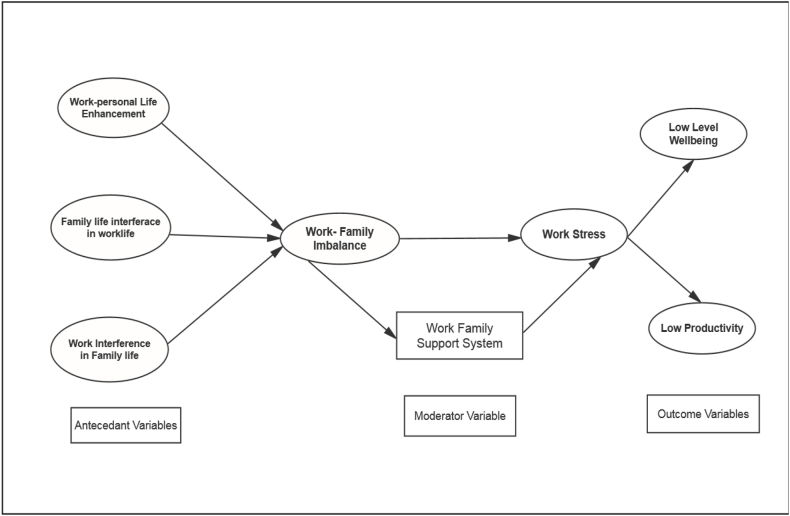
Research model.

**Fig. 2:**
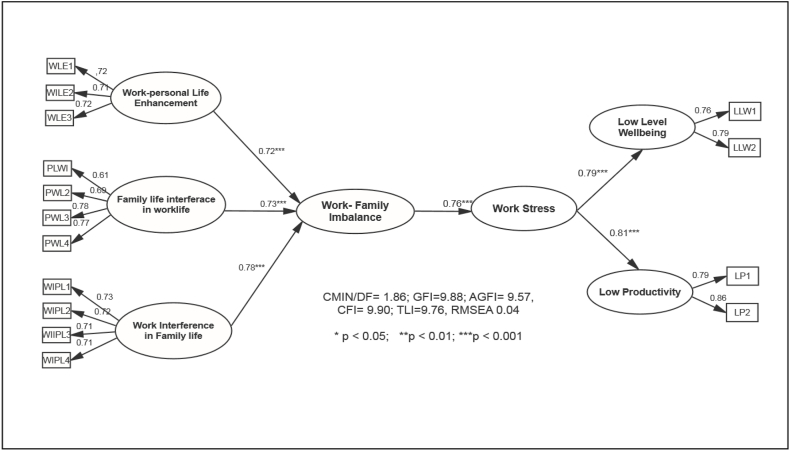
Complete SEM model testing the hypothesized effects of work-life balance on work stress.

**Fig. 3:**
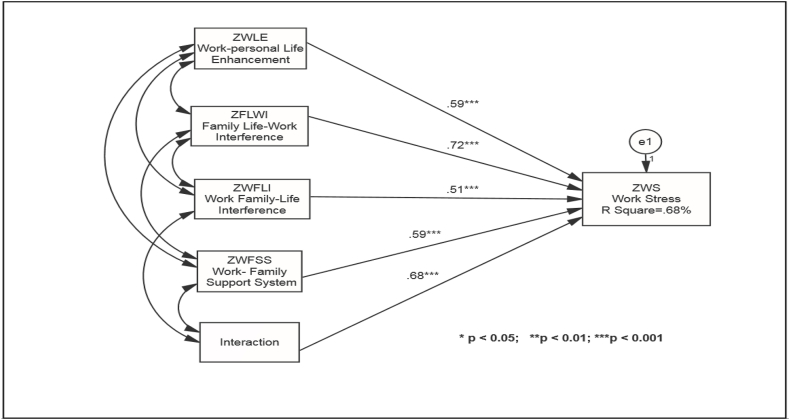
SEM models testing the moderating effect of the work-family support system on work stress.

Similarly, the formative model tested if the work stress is reflected through low levels of well-being and increased anxiety. The hypotheses related to the all-causal measurements were also confirmed (0.79; 0.81, p < 0.001). Finally, a formative measure tested the causal effect of work-life balance on work stress. The hypothesis predicting work-life balance's impact on work stress showed a significant causal effect (0.76, p < 0.001). The complete research model analysis in Fig. 2 supports all the hypotheses, as the path model shows a good model fit. The default model in the figure shows that the model fit indexes meet the established benchmarks suggested by Hu and Bentler (1999). Fig. 1 shows the default model scores (*χ2 (214) = 186.02, p* < *0.01; GFI = 9.88; AGFI = 9.57; CFI = 0.990; TLI = 0.976; RMSEA = 0.04).* Since the model scores were good, no alternative model was tested in this study (see Fig. 3).

Further, the authors tested the moderation effect of the work-life support system on work stress. The objective was to examine if such support systems can reduce the intensity of work stress. Fig. 2 shows that the interaction effect of the work-life support system on work stress is significant (0.68, P < 0.01). It implies that work-life support systems such as work-life support policies, supervisory support, and flexible accommodations can reduce work stress's impact on employees' anxiety levels and well-being.

The author accepts all the hypotheses based on the results of the SEM path model and moderation model. Table 3 shows the results of the hypothesis testing.

## 6 Discussion

The study examined the drivers of the work-life imbalance of women administrators and the adverse effects on their personal and professional lives. The proposed research model illustrating the inter-role experiences of women administrators adds to the extant literature's efforts to contextualize work-life imbalance antecedents and offer solutions to reduce the effect of work-life imbalances. It is one of the first models that can be applied to study the work-life balance issues of women professionals in HEIs in the current research setting. The study argued that the antecedents of work-family imbalance for women administrators should be studied in the social context, such as family goals, career aspirations, cultural compulsions, and organizational support systems. These drivers of work-life imbalance become even more relevant in the context of women administrators as their work lives are significantly intertwined with their family lives, considering they play the dual role of homemakers and professional employees.

The study discovered that women administrators’ family lives suffer due to increased work responsibilities and pressure to meet social and cultural expectations. On the one hand, they want to advance their careers through job enrichment, but on the other hand, the demand for personal commitments creates a conflict and a work-family imbalance. Work-life enhancement aspiration impels women administrators to put extra effort at work, while family-life enrichment goals pull them apart from work commitments, leading to work-family imbalances. The study noted that aspirations rise as women's careers progress, and they begin to invest more time, energy, and emotional resources at work to realize their career goals. If there is no support from family and organization, the spillover of work in the family domain leads to work-life imbalances. Based on the findings, the study accepts the first hypothesis (H1) that posited that increased work-life enhancement aspirations lead to work-life imbalances.

Women are attracted to academia due to the cliched perception of satisfying and comparatively low-stress jobs. However, the growing pressures of researching, teaching, and administrative work in academia have forced administrators to work overtime and weekends, either on or off campus. The study in the current context found that role overload of women administrators limits their ability towards family care, time with friends, and time for self-care. Further, female employees, compared to men, invest more emotional energy in their family and work lives, creating imbalances and inconsistencies in allocating resources such as time and energy to work or family. Based on the findings, the study accepts the second (H2) and third (H3) hypotheses that increased work pressure among women administrators is a significant cause of work-family life imbalance. When emotional investments are high, women tend to get more stressed as they cannot quickly get psychologically detached, leading to work stress. This study found it true in the current research context, leading to the acceptance of research hypothesis four (H4). The spillover of feelings, emotions, and behaviors between job and life leads to work stress, usually manifested as anxiety and mental and physical ill-being.

The work-family support system is a critical driver acting as a moderator in this complex work-family tension. The work-life imbalance is not just about work and family conflict; it's about gender equality, decent work for women, good health, and well-being. Therefore, it is more of an organizational responsibility to institute work-life balance support systems. The study found that work-family balance support systems for women administrators can moderate the effect of work-life imbalance drivers on work stress in the current research setting. Creating a work-family support system enables the integration of work-family life and balances the tensions between the two. It also cushions against spillovers over work and family commitments. In other words, when better and more organized corporate support systems exist, role imbalance and spillovers are reduced, leading to a weaker effect of work-life imbalance. Based on these findings, the study accepted hypothesis five (H5), which postulated the moderating role of organizational support systems on work-family life imbalances.

### 6.1 Key findings concerning the broader literature

The results of this study support previous research on work-life imbalances and found that some of the antecedents and outcomes of work-life imbalance of women in HEIs were similar to other professional contexts. These findings have also been highlighted in studies conducted by Pan and Sun [88] and Wayne et al. [89], who explained the difficulties faced by women who balance homemaking and career aspirations.

This study, building on the work of Ahmed and Asfahani [90], Jabeen et al. [30], and Miznah [54], further argued that societal expectations further burden women administrators in higher education institutions, making it even more challenging to balance their roles. The findings align with the findings of Taylor et al. [33] and Kang and Kaur [91], who argued that women's aspirations increase as they progress in their careers. This results in a more significant time, energy, and emotional investment required to achieve their goals. Furthermore, the findings of this study resonate with Bartlett et al. [92] and Bothwell [93], who found that work-life imbalances in academia spill over to family life, impacting academics and their organizations' well-being.

This study contributes to the extant literature by highlighting the natural spillovers of work and family life and the clash between professional aspirations and personal family and social commitment set against quantitatively and qualitatively demanding work in HEIs in the region. Further, it proposes work-family imbalance solutions. The study, supporting the arguments of Anastasopoulou et al. [94] and Agha [85], emphasizes that addressing work-family life imbalances is crucial to promoting gender equality, ensuring decent work for women, and promoting good health and well-being. Therefore, it is primarily the responsibility of organizations to ensure work-family life balance by designing work-life balance support systems. The objective of WLB can be achieved through building an organizational culture of trust, learning, and commitment [14,70].

## 7 Theoretical implications

Most of the research on the spillover theory has focused on either the negative or positive effects of spillovers or the outcomes, such as stress and ill-being due to the spillover of work and family life [95]. The Spillover theory appropriately captures the complexity and uniqueness of work-life imbalances of women administrators in HEIs. The study explained that spillovers result from rigorous work requirements and personal ambitions. As a result, the study posited that these complex spillovers contain elements of personal, social, and emotional undercurrents and are not straightforward relationships. Therefore, a bipolar view of spillovers must be avoided when applying the theory to industry and social contexts. Further, the study found the moderating role of organizational support systems in facilitating work-life balance effectively.

The study also contributed to the facilitation theory, expanding its application to organizational learning. Organizational learning is possible within work-life balance support systems to build cultures of self-learning, adaptation, flexibility, and self-awareness. The study sheds light on how organizations can facilitate empowerment toward work-life decisions, build trust, and create a collective culture of work-family balance among women. Cross-pollinating the spillover theory with the facilitation theory helped create a problem-solution model research framework that can be further extended and explored.

### 7.1 Practical implications

The findings imply that female administrators need better work-life balance support systems to ensure they are not stressed at work and are not disadvantaged as against their male counterparts. Thus, the research model developed and tested in this study has a higher applicability in HEIs for analyzing women's work-family balance than a general model of work-life imbalance. It is evident from the findings and the discussion that the HEI women administrators need appropriate work-family support systems for sustainable employment, high-performance work, increased employee morale, and practice innovation at work. The implications of the findings reinforce the merits of work-family balance that are both beneficial to the employees and the employers. The WLB policies and accommodating working practices of women administrators in HEIs support the organization in lowering work-family imbalances and stress. Organizations that provide sound work-family support systems to their employees experience increased competitiveness and productivity, leading to improved customer service [96].

Hybrid working models, one of the prominent WLB strategies, support the organization in adapting to the dynamic market demand and accommodating family life commitments. Shift working, part-time working, and flexi-time working enhance the hybrid working culture to offer customers extended service hours without demanding the employees to work for longer hours. In addition, such working arrangements boost employee morale, commitment, and a positive attitude toward work [97]. One of the most favorable outcomes of a work-family support system is reduced work stress and employee turnover, which helps the organization minimize recruitment costs. Also, employer branding can be established through sustainable work-life support systems, attracting highly talented employees wanting to work for WLB-supportive organizations [98]. The findings demonstrated that work-life balance and role human resource practices positively affect women managers' well-being. Women administrators in HEIs enjoying an excellent work-life balance feel valued and reflect the same with their family and social community.

## 8 Limitations and future research directions

One of the significant limitations of this research is that the study used cross-sectional data to analyze antecedents and outcomes of work-life imbalances. The causal relationships could have been stronger with cross-lagged data. Another limitation of the study was that it considered the broader family and social life domain to assess work-life imbalances, creating a wide canvas of spillover possibilities rather than focusing on the family domain. Finally, the study did not analyze if social arrangements at the workplace could compensate for some of the low emotional well-being and stress at work. Therefore, future research could utilize longitudinal data to investigate the effect of social workplace involvement and professional engagement in balancing work-life imbalances.

## 9 Conclusion

The study concludes that work-life imbalances persist despite substantial efforts to reduce them. Work-family life imbalances are rooted in social and family structures and associated values. Therefore, the context of the research setting and sector-specific antecedents and outcomes of work-life imbalances are critical to understand. The work-life imbalance reflected through family interference in work-life and work-life interference in family life among women administrators in the higher education sector is driven by the nature of work and professional aspirations as they tend to spill over on each other domains. Administrative oversight and research require women administrators' commitment and involvement beyond duty hours; hence, imbalances occur. Since the effects are detrimental to women administrators and their organizations, efforts to reduce the impact should be continuous, as the measures have not shown the desired results. Organizations should continue to explore effective human resource practices to moderate the effect of work-life imbalances. It is pivotal for organizations to exercise hybrid work arrangements embedding technology and WLB support systems in supporting the improvement of employee motivation and performance and reducing work stress.

## Data availability statement

Data will be made available on request.

## CRediT authorship contribution statement

**Vazeerjan Begum:** Visualization, Investigation, Formal analysis, Data curation. **Tahseen Anwer Arshi:** Visualization, Supervision, Resources, Formal analysis. **Abdelfatah Said Arman:** Writing – review & editing, Validation, Software, Resources, Methodology, Conceptualization. **Atif Saleem Butt:** Writing – original draft, Visualization, Validation, Data curation, Conceptualization. **Surjith Latheef:** Visualization, Software, Methodology, Investigation, Formal analysis.

## Declaration of competing interest

The authors declare that they have no known competing financial interests or personal relationships that could have appeared to influence the work reported in this paper.

## Figures and Tables

**Appendix 1:** Data characteristics.

Variable	(n = 274)
Gender	F
Highest Education Qualification (Undergraduate/Graduate)	(U = 161) (G = 113)
Type of Higher Education Organization (Private/public)	(Pri = 55) (pub = 23)
Years of Administration Experience (less than 5 yrs/more than 5 yrs)	(<5yrs=89) (>5yrs = 185)
Age Group (25–34) (35–44) (45–54) (55–64)	(25–34) = 21 (35–44) = 124 (45–54) = 94 (55–64) = 35
Local or Expatriate	L = 109 E = 165
